# Disadvantages of red: The color congruence effect in comparative price advertising

**DOI:** 10.3389/fpsyg.2022.1019163

**Published:** 2022-11-17

**Authors:** Heejin Kim, Jung Min Jang

**Affiliations:** ^1^College of Business, Gachon University, Seongnam, Gyeonggi, South Korea; ^2^College of Business, Arts and Social Sciences, Brunel University London, Uxbridge, United Kingdom

**Keywords:** color congruency, discount depth, comparative price advertising, processing fluency, value perception

## Abstract

Based on the metaphorical association of color, the color red is often associated with a “hot deal” in a price promotion context, which has led to the popular use of red in promotions in the marketplace. In two studies, this research challenges the lay belief that the color red used in price advertising positively influences consumers’ perceptions of value. The findings from the two studies revealed a contrasting pattern of results depending on the depth of the discount. When the discount depth was high (consistent with consumers’ lay belief), the red color led to more favorable responses to a price promotion than other colors (white or blue in Study 1 and green in Study 2). In contrast, when the discount depth was unambiguously low, consumers who were exposed to a red colored price promotion advertisement reported a perception of a lower value compared to those who saw the same advertisement with other colors We attribute this effect to the degree of “processing fluency” that arises from the congruence between the color and promotion content. Our research adds to the existing psychology literature on color functioning by showing a match between the referential meaning of the color red (i.e., a hot deal) and consumers’ perceptions in marketing communication.

## Introduction

A price discount is one of the most widely used marketing techniques retailers employ to increase sales. An analysis of panel data collected on 30,000 British shoppers showed that 40% more food and drink purchases were made when the items were on sale (Public Health England, 2020). Similarly, Sheehan et al. (2019) audited the websites of the largest online grocery retailers in the United States and found that 37% of the products were advertised in the store’s discount price promotion. According to prior research, the attractiveness of a discount promotion is determined by consumers’ perception of the magnitude of the discount (Kahneman and Tversky, 1979; Kalyanaram and Winer, 1995). Several studies have demonstrated that the perceived magnitude of the discounts are affected by the visual context in advertising such as the size (Coulter and Coulter, 2005; Aggarwal and Vaidyanathan, 2016) and font style of the discounted prices (Motyka et al., 2016; Park and Ryu, 2019), location of the discount information (DelVecchio et al., 2009; Jang and Park, 2020), distance between the original and discount prices (Coulter and Norberg, 2009) and the color of the text or background (e.g., Labrecque et al., 2013; Puccinelli et al., 2013; Ye et al., 2020). Extending previous research, the current study investigated the role of the color red in consumers’ evaluations of a price discount promotion.

Most prior research on color in price promotions has been based on the conventional wisdom that using the color red helps build consumers’ perceptions of a discount (e.g., Labrecque et al., 2013; Puccinelli et al., 2013). Thus, retailers often use the color red in price promotion advertisements. For example, Amazon indicates a discounted price in red while keeping the list price in black, and Walmart often uses a red background for flyers to announce price promotions. However, contrary to conventional wisdom, the current research suggests that the use of the color red may backfire if the depth of the discount is lower than what consumers generally believe is “great savings.” Our prediction is underpinned by the considerable research highlighting the role of congruence among the central content and visual cues in marketing communication (Heckler and Childers, 1992; Peracchio and Meyers-Levy, 2005; Van Rompay et al., 2010). According to this stream of literature, congruence among the meaning of various elements in advertising (e.g., verbal information and visual cues) enhances persuasion, whereas incongruence may engender disfluency and lower the attractiveness of a target object (De Bock et al., 2013; Wang et al., 2020). Similarly, we expect that conceptual congruence between the depth of the discount and the color red in an advertisement can affect the attractiveness of the deal. In particular, if the discount depth does not match the meaning that the color red symbolizes (high discount or great savings), consumers may experience disfluent processing, which can lead to unfavorable responses.

The current study makes several contributions to the academic field. First, we extend the literature on color effects in consumer psychology (see Labrecque et al., 2013, for a review). Considerable research has examined the role of the color red in various marketing contexts, yet only a handful of studies have been conducted in a price-related context. Moreover, while the majority of studies have shown the beneficial side of using color to enhance the intended communication, the current study is among the first to assess the detrimental effect of using colors (particularly red) that are commonly used to highlight a particular meaning. This research also builds on the price promotion literature, which highlights the importance of contextual factors (Grewal et al., 1996; Koo and Suk, 2020) by adding a new variable that regulates the perceived value of the deals. In particular, we focus on the influence of color when retailers provide a specific reference price (i.e., comparative price promotion setting). We show that the match between the symbolic meaning of the color and the discount depth can increase but also decrease the attractiveness of the comparative price promotion.

## Theoretical background

### The color red in price promotions

Color is ubiquitous in human communication (i.e., Fugate and Franco, 2019; Schlintl and Schienle, 2020). As such, a sizable body of research has demonstrated that the colors used in advertising influence consumers’ perceptions, judgment, and behavior in various domains (Bellizzi and Hite, 1992; Meyers-Levy and Peracchio, 1995; Lichtlé, 2007; Mehta and Zhu, 2009; Seo and Scammon, 2017). A common denominator among these findings is that color conveys specific meaning and information (Elliot et al., 2007). According to this stream of literature, when people are repeatedly exposed to a particular color paired with a particular message, they form cognitive links between the color and the paired concept (Elliot et al., 2007; Labrecque et al., 2013). As a result, mere exposure to a specific color triggers the conceptual association, which influences consumers’ perceptions. For example, a red color used for traffic lights or warning signs is associated with “stop” or “danger” and triggers avoidance motivation for consumers (Mehta and Zhu, 2009). Similarly, green infers the natural environment, so consumers perceive that the product as nature-friendly (Seo and Scammon, 2017).

According to color-in-context theory (Elliot and Maier, 2012), color conveys different meanings in different contexts. For instance, the color red signals mistakes or failure in an achievement context (e.g., academic classes) whereas the same color may involve heterosexual attractiveness in an affiliation context. In the price promotion context, a red tag infers that the items are on sale (e.g., a red-tag sale). In this context, the color red is generally associated with attractive deals with deep discounts. Considerable anecdotal evidence on the color red signaling deals with great savings supports this notion (Ye et al., 2020). Discount stores highlight the offered price in red and retailers often use the word “red” to refer to special deals (e.g., “Vodafone Red” for a value payment plan from Vodafone or redflagdeals.com, an online discount coupon site). Findings from previous studies also support these market practices and consumers’ perceptions. For example, a content analysis of 500 print flyers from major retailers in the United States revealed that retailers that emphasize a value image (e.g., Walmart and Target) use red to indicate pricing information and savings (Puccinelli et al., 2013). Labrecque et al. (2013) suggested that people are likely to associate prices in red with discounts. Similarly, Puccinelli et al. (2013) demonstrated that male shoppers use red prices as visual heuristic cues to judge the savings and thus evaluate prices in red (vs. black) more favorably.

Notably, previous research showing the positive effects of the color red in consumers’ perceptions of savings have been tested mostly with stimuli that only provided the final (promotion) price without indicating the original price or the discount depth, or information on the amount of savings from the promotion was rather ambiguous. However, these contextual cues may be the basis for consumers to infer the attractiveness of a deal. When concrete information about the discount depth is provided, the actual discount depth can be compared with what the contextual cues symbolize. Consequently, consumers’ judgment and evaluation can be influenced by whether the actual discount depth matches the representation of the color red. In the next sections, we introduce comparative price promotion advertising in which both the original and discount prices are indicated and show how consumers process concrete discount information for a price promotion evaluation.

### Discount depth in a comparative price promotion

The attractiveness of a discount promotion is highly dependent on consumers’ subjective judgment of the discount depth (Kahneman and Tversky, 1979; Della Bitta et al., 1981; Gupta and Cooper, 1992; Kalyanaram and Winer, 1995; Kukar-Kinney and Carlson, 2015). According to prior research, the consumers’ perception of the discount depth is formed by comparing the current price (i.e., the discount price) and a reference price (i.e., the original price; Berkowitz and Walton, 1980; Kalyanaram and Winer, 1995). Although consumers’ internal reference based on the original price they observed is an important determinant (Kalwani et al., 1990; Mazumdar et al., 2005), much research has also shown the impact of external information in the formation of the reference price. Even irrelevant contextual cues can affect consumers’ price estimation and judgment of the magnitude of the discount (Thomas and Morwitz, 2009). For instance, Nunes and Boatwright (2004) showed that the advertised price of unrelated goods on display can alter consumers’ valuation of a product. Similarly, Adaval and Monroe (2002) demonstrated that exposure to high and low numbers in an irrelevant dimension (e.g., weight) affects consumers’ price evaluation at the subliminal level.

Among other external information, retailers frequently display both the original or previous price so consumer can compare it with the discounted price to directly influence buyers’ reference price (Della Bitta et al., 1981; Allard and Griffin, 2017; Feng et al., 2017). This comparative price promotion tactic (Grewal et al., 1998; Choi and Coulter, 2012) typically displays the regular or original price as a reference price so consumers can contrast it with the promoted price and infer that they will save money (Compeau and Grewal, 1998). With this additional price information, consumers can calculate the depth of the discount and compare the current price with the reference price provided by retailers to guide their value judgment (Feng et al., 2017). Studies have also shown that a comparative price promotion generates a higher willingness to pay compared to a promotion in which only the sale price is displayed (Della Bitta et al., 1981; Compeau and Grewal, 1998; Anderson and Simester, 2009).

A distinctive characteristic of a comparative price promotion is the level of perceived ambiguity in the depth of the discount. In a non-comparative price promotion context (i.e., without indicating the original prices or discount depth), the amount of savings from the promotion is rather ambiguous (Licata et al., 1998), so consumers need to make inferences based on non-diagnostic cues such as colors. Naturally, individuals are likely to assimilate their judgment to the meaning that the contextual cues symbolize (Herr, 1989; Stapel et al., 1997). By contrast, in a comparative price advertisement, consumers can calculate the specific discount depth (Grewal and Compeau, 1992). If the discount depth is estimated in the consumer’s mind with no ambiguity, it may generate a contrast effect in the opposite direction to the presented non-diagnostic cues under certain conditions. Specifically, consumers’ perception of the depth of a concrete discount can be represented as a kind of category membership, such as a low or high discount. When the features of a particular discount depth category and the associated semantic meaning of the non-diagnostic cues do not match, the non-diagnostic cues are unlikely to serve as converging information for the discount depth judgment (Herr et al., 1983; DeCoster and Claypool, 2004). As such, we believe that the discount depth and value-signaling color within a comparative price advertisement can have a congruence effect.

### Congruency effect: Relationship between discount depth and the color red

The theoretical foundation for the current research stems from the literature on conceptual congruence. Congruence refers to entities that go well together (Maille and Fleck, 2011). According to prior research, typical sources of congruence are conceptual as well as emotional similarities (Maille and Fleck, 2011). In the current research, we propose that the two main constructs, discount depth and the color red, are conceptually similar, which cultivates congruency. When people make sense of an abstract concept, they utilize a concrete concept in their memory (e.g., Clark, 1973; Jang and Park, 2020). In a price promotion context, the color red is likely to be associated with “great savings.” As a result, when discount information is presented in red in comparative advertising, we can expect a compatible relationship between the color and the discount depth, which fosters (in)congruence.

Much prior research has suggested that congruence of meanings expressed across and within marketing communication elements enhances consumers’ evaluations (Heckler and Childers, 1992; Van Rompay et al., 2009, 2010). For example, Peracchio and Meyers-Levy (2005) showed that congruity in symbolic connotations in images and slogans in an advertisement led to a favorable attitude about the product. More recent studies on comparative price advertising have demonstrated that the match between the discount depth and non-diagnostic information such as physical proximity, font size, and sound can moderate or influence consumers’ responses to the information (e.g., Coulter and Coulter, 2005; Coulter and Norberg, 2009). These studies support our conjecture by showing that assimilation between the price perception and non-diagnostic peripheral information occurs only when the mental representation of the provided information (i.e., discount depth) is compatible with the characteristics of the non-diagnostic information. For instance, Coulter and Coulter (2005) examined how the interplay of the magnitude representations related to the prices and font size affected consumers’ attitudes toward a price promotion. Specifically, when consumers viewed advertisements in which the font size of the prices were presented congruently with their magnitude representations (e.g., a larger font is used for the regular price and a smaller font for the sale price), consumers had more favorable attitudes toward the offered price. Coulter and Norberg (2009) showed that a greater spatial distance between the two prices (sale and regular prices) led to the perception of a greater difference, resulting in a more favorable attitude toward the amount of savings in the deal.

Several studies have also reported that incongruence among elements in a single stimulus may generate negative responses (i.e., Coulter and Coulter, 2005; De Bock et al., 2013). For instance, Coulter and Coulter (2005) found that when the font size was incongruent with the presentation of the price magnitude (e.g., a larger font for the sale price vs. a smaller font for the regular price), consumers had less favorable attitudes. De Bock et al. (2013) demonstrated that mismatching color cues with the message could have a detrimental effect on people’s moral judgment. They presented participants with a statement about (un)ethical behaviors written on a red (immoral color) or green (moral color) background and measured the desirability of the statement. The results showed that when immoral behavior is presented on a green background, the perceived desirability of the statement decreased. Their findings suggest that even a color that is commonly perceived as positive (e.g., green) can have a negative influence when the meaning does not match the information.

Extending these prior findings, we expect that consumers will have a favorable perception of a price promotion only when the information (in this context, the gap between regular and sale prices) is congruent with the meaning of the color red (great savings). Specifically, we predict:

*H1 (Moderation)*: The discount depth will moderate the effect of the color red on the attractiveness of the deal in a comparative price advertisement.

*H1a*: In a high discount depth condition, consumers will show increased deal attractiveness toward the price promotion when using the color red (vs. other colors) in the advertisement.

*H1b*: In a low discount depth condition, consumers will show lower deal attractiveness toward the price promotion when using the color red (vs. other colors) in the advertisement.

### Underlying mechanism: Fluency as a mediator

In the current research, we posit that congruency between the discount depth and the color will regulate consumers’ processing fluency, which, in turn, will influence promotion attractiveness. Processing fluency refers to the ease with which consumers can process the information (Lee and Aaker, 2004). According to the fluency literature, people misattribute the positive feelings elicited from fluent processing to the stimuli being processed rather than to the ease of processing (Winkielman et al., 2003). As a result, a fluent processing experience enhances the effectiveness of persuasion (Lee and Aaker, 2004; Labroo and Lee, 2006).

Extensive research has demonstrated that conceptual congruence of information that is presented together increases individuals’ processing fluency (Lee et al., 2010; Chae and Hoegg, 2013; Kareklas et al., 2019; Wang et al., 2020). For example, Boot and Pecher (2010) showed that congruency between the physical distance of colored squares (i.e., near vs. far) and the similarity of the color (similar, dissimilar) influenced people’s reaction time and error rates in a similarity judgment task. In the advertising and marketing field, the relationship among conceptual congruence, processing fluency, and consumer responses has been well-documented (Lee et al., 2010; Kareklas et al., 2019; Wang et al., 2020). For instance, Chae and Hoegg (2013) showed that people evaluate furniture more favorably when the attributes (antique vs. modern) match the spatial representation of time (past on the left vs. future on the right) and the effect is mediated by processing fluency. A similar effect has also been reported in the area of color research. For example, using a green background color for environmental claims (Seo and Scammon, 2017) or matching a pink color with research on breast cancer (Kuo and Rice, 2015) increased behavioral intention by increasing fluency.

Extending these lines of research to the comparative price advertising context, we posit that the congruency between the discount depth and the color in the advertisement can be a source of processing fluency and consequently influence promotion attractiveness. Specifically, we posit that when the discount depth is high, people perceive that the color red is congruent with the content of the promotional offer (i.e., discount depth). As a result, they experience enhanced processing fluency and, in turn, have a positive attitude toward the promotion. However, when the discount depth is low, the referential meaning of the color red (great savings) and the discount depth are incongruent. Such conceptual incongruence may create disfluency in processing, which will negatively affect consumers’ evaluations. Thus, we predict:

*H2 (Mediation)*: Processing fluency will mediate the relationship of the interaction between the discount depth and the color (red vs. others) and the deal attractiveness of the advertised promotion.

## Studies

### Pretest: Association test between the color red and the discount depth

Our prediction is based on the assumption that people strongly associate the color red with “great savings.” Thus, before exploring the hypothesis, we attempt to determine whether there is an association between the color red and the perception of value (i.e., attractive deal) compared to other colors in comparative price advertising.

Procedure: A pretest (*n* = 32) was conducted with college students. All participants were presented with an online coupon book containing discount coupons for various product categories. Then, they were informed that by clicking the icon, a cover page and each coupon would be displayed sequentially. As the cover page appeared on the screen, participants were asked to indicate their expected value of the coupon (i.e., “the savings from the promotion will be high,” “the value of the promotion will be good”). Depending on the condition, the background color of the cover page differed (red, green, or white). All letters on the cover page were written in the color gray.

Results: A comparison of the three backgrounds revealed that the color red accentuates the promotion deal value. Specifically, participants exposed to the red background (*M_red_* = 4.45) expected the savings to be higher than those exposed to the green background (*M_green_* = 3.23, *F*(1,19) = 1.81, *p* = 0.05) or the white background (*M_white_* = 3.32, *F*(1,19) = 6.71, *p* = 0.02). These results provide supportive evidence that the color red is strongly associated with great savings.

### Study 1: Color congruence effect

Study 1 explores the congruence effect of the color red with the discount depth in comparative price advertising (H1 (Moderation)). We predict that participants will have lower perceived value of the promotion in the advertising using the color red (vs. white or blue) under a low discount condition, which is incompatible with the meaning of the color red (i.e., hot deal). We expect that participants will show the opposite pattern in a high discount condition, which is compatible with the meaning of the color red.

#### Materials and methods

##### Participants and design

A total of 185 undergraduate students (43.8% female, *M_age_* = 21.67, *SD_age_* = 2.29) were randomly assigned to the conditions with a 2 (discount depth: high vs. low) × 3 (background color: red vs. white vs. blue) between-subjects experimental design. Participants voluntarily participated in the study and completed the survey in exchange for extra course credit.

##### Stimuli and procedures

We considered several aspects when selecting the color to create the experimental conditions. First, we chose the color white because it is one of the most typical background colors when presenting prices in marketing communication. Thus, a white background was used for the control condition. We next added the color blue as another control condition. Information with certain colors attracts respondents’ attention and consequently amplifies their attitude about the advertising and the offered product (Sparkman and Austin, 1980). Similarly, the colored information may heighten the value perception in the high-discount condition and lower it in the low-discount condition. Thus, the expected result could be driven by the colored (vs. white/non-colored) background if there were only two conditions (red vs. white). Using the color blue, which is not associated with savings in a price promotion and is a different-colored stimulus, strengthens our rationale that the expected effect is only caused by the color red when the proposed effect is observed.

Prior literature has suggested that when the discount depth is not provided, consumers expect ~10%–12% savings (Blair and Landon, 1981; Biswas et al., 2013). Reflecting consumers’ general expectation, we considered a 10% or lower discount depth as a “low discount” and 30% or more as a “high discount.” The discount offer was printed in black letters, with the background colors of either red, white, or blue manipulated for the various conditions (see Appendix A).

All participants were presented with a print advertisement featuring the announcement of a promotion offering a price discount for a foreign language learning program. In the high-discount condition, a US$45 discount (30% discount from full price) was offered, whereas in the low-discount condition, a US$7.50 discount (5% discount from full price) was offered. Once participants read the advertisement, they rated the perceived attractiveness of the promotion (“The discount from the promotion is high,” “The value of the promotion is good”; Cronbach’s *α* = 0.93) on a 9-point scale (1 = do not agree at all to 9 = agree very much). Then, to meet the general expectation of the promotional offer in the category, participants were asked to report the discount rate they usually observed in similar language learning programs.

#### Results

##### Manipulation check

The participants reported that the general price discount offered in a similar product category was 16.63% (*SD* = 22.66), on average. The 5% (30%) discount offered in the current research was perceived as unambiguously lower (higher) than people’s general expectation of the category discount. A one-sample *t*-test at a test value of 5% revealed that the 5% discount was significantly lower than respondents’ expectation regarding the usual discount rate in the category (*t*(144) = 6.18, *p* < 0.01), while the 30% discount condition was significantly higher than respondents’ general expectation (*t*(144) = −7.12, *p* < 0.01).

##### Promotion attractiveness

**Figure 1:**
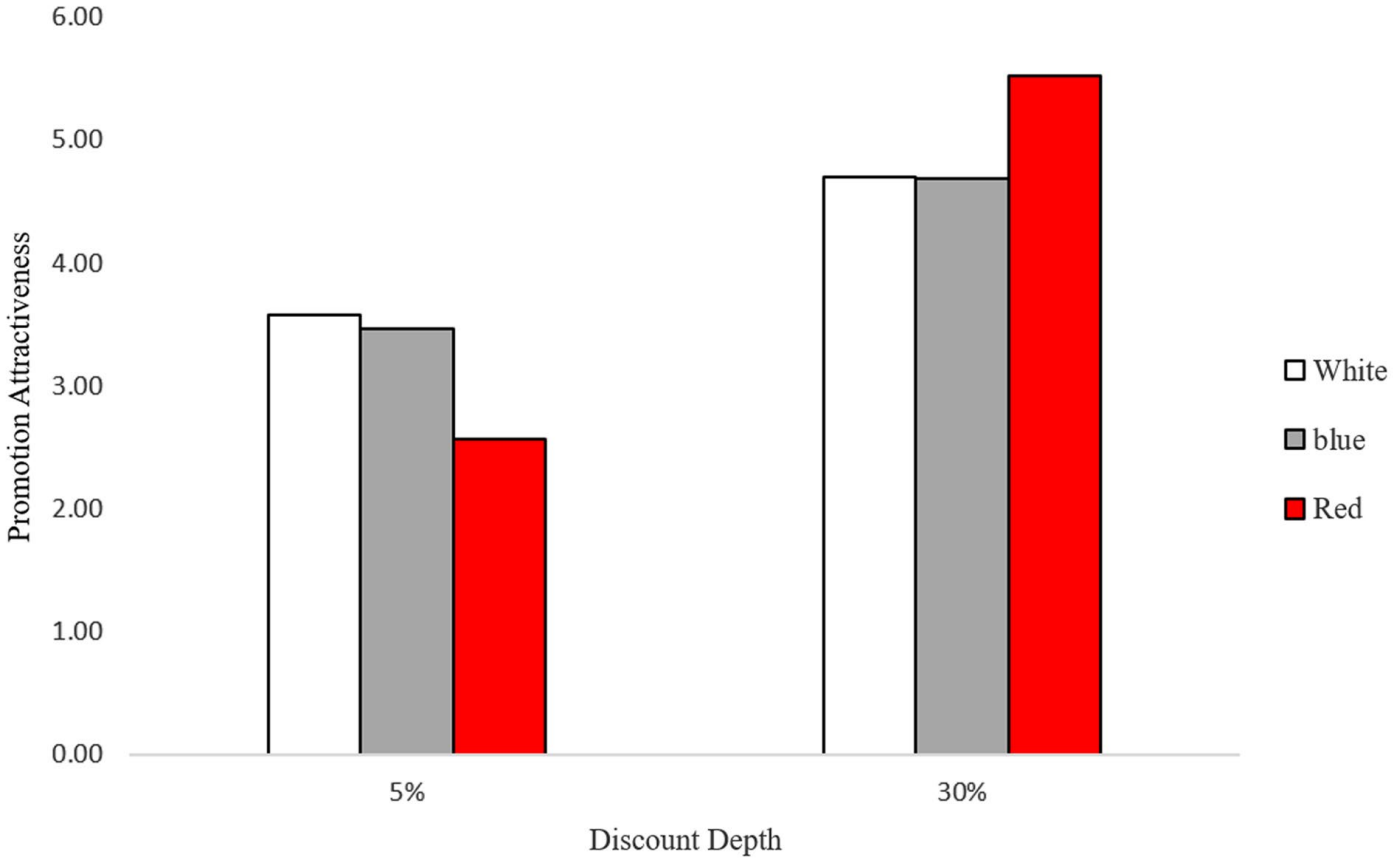
Promotion attractiveness as a function of background color and discount depth.

To analyze the effect of color and discount depth on the attractiveness of a price promotion, a two-way analysis of variance (ANOVA) was performed. As expected, the results revealed a significant main effect of the discount rate (*F*(1,179) = 55.43, *p* < 0.01) on the perceived value of the promotion. More importantly, a significant interaction effect between the background color and discount depth was also observed (*F*(2,179) = 6.21, *p* < 0.01). To provide pairwise comparisons, further contrast analysis was employed. When the discount rate was high (30%), the perceived value of the promotion was higher with the red background (*M* = 5.52, *SD* = 1.48) compared to the white background (*M* = 4.70, *SD* = 1.83), (*F*(1,179) = 3.95, *p* = 0.05) or the blue background (*M* = 4.69, *SD* = 1.56), (*F*(1,179) = 4.11, *p* = 0.04). Conversely, when the discount rate was low (5%), the value of the promotion was perceived as lower with the red background (*M* = 2.57, *SD* = 1.25) than with the white background (*M* = 3.58, *SD* = 1.91), (*F*(1,179) = 5.98, *p* = 0.02), or the blue background (*M* = 3.47, *SD* = 1.53), (*F*(1,179) = 4.78, *p* = 0.03). Additionally, there was no difference between the white and blue background conditions in both discount conditions 30%: (*F*(1,179) = 0.002, *p* = 0.97); 5%: (*F*(1,179) = 0.08, *p* = 0.78; see Figure 1).

#### Discussion

Consistent with our expectation, the result of Study 1 provides evidence that supports the color congruence hypotheses. When the savings from the promotion were lower than the expectation, participants perceived lower attractiveness to the promotion advertisement with the red background compared to the other colors (i.e., white or blue), whereas the opposite pattern was found in the high-discount condition.

### Study 2: Mediating role of “processing fluency”

Study 2 extends the previous investigation with several meaningful goals. First, we attempted to replicate the results by changing the font color instead of the background color because different font colors are commonly used in price promotion advertising to convey price information. In addition, we used a different color (i.e., green), and a different product (i.e., Bluetooth speaker) to strengthen the generalizability. Secondly, to understand the underlying mechanism of the proposed effect, we measured the sense of “processing fluency” and tested the mediating role.

#### Materials and methods

##### Participants and design

A total of 123 participants (36.6% female; *M*_age_ = 35.41, *SD_age_* = 13.07) recruited from Amazon Mechanical Turk (MTurk) were randomly assigned to the conditions with a 2 (discount depth: high vs. low) × 2 (font color of price presentation: red vs. green) between-subjects design. Participants voluntarily participated in this study and completed the survey in exchange for a small monetary reward.

##### Procedures

All participants were presented with an image on the computer screen of an advertisement that promoted the discounted price of a Bluetooth speaker. The image was designed to look like a real online advertisement and included the regular and sale prices of the product as well as the discount depth. A product picture and a short description were also presented regardless of the experimental condition. In the high-discount condition, the regular price was 58 thousand Korean Won (TKW; US$51.40), and the sale price was 34.8 TKW (US$30.84; ~40% discount from full price), whereas in the low-discount condition, the sale price was 56.26 TKW (US$49.85; ~3% discount from full price). The discount offer was printed in red or green letters, while the background color was white for both color conditions (see Appendix B). Once participants read the advertisement, they were asked to indicate their perceived attractiveness to the promoted offer (Cronbach’s *α* = 0.95). Then, the sense of “processing fluency” was measured with four items that asked participants to rate their thoughts or feelings while reading the advertisement on a 7-point scale (1 = not at all to 7 = very much). The items included “It was hard to understand/It was easy to understand”; “It was hard to process the info/It was easy to process the info”; “It was not at all organized/It was well organized”; “It was not at all structured/It was well structured” (adopted from Chae and Hoegg, 2013; Cronbach’s *α* = 0.91). Lastly, we measured their mood, knowledge, and future purchase plan as control variables to explain potential internal validity threats. The control variables were not correlated with the interactions of the font color of the price presentation and discount depth. Thus, we excluded these variables for the following analysis.

#### Results

##### Promotion attractiveness

**Figure 2:**
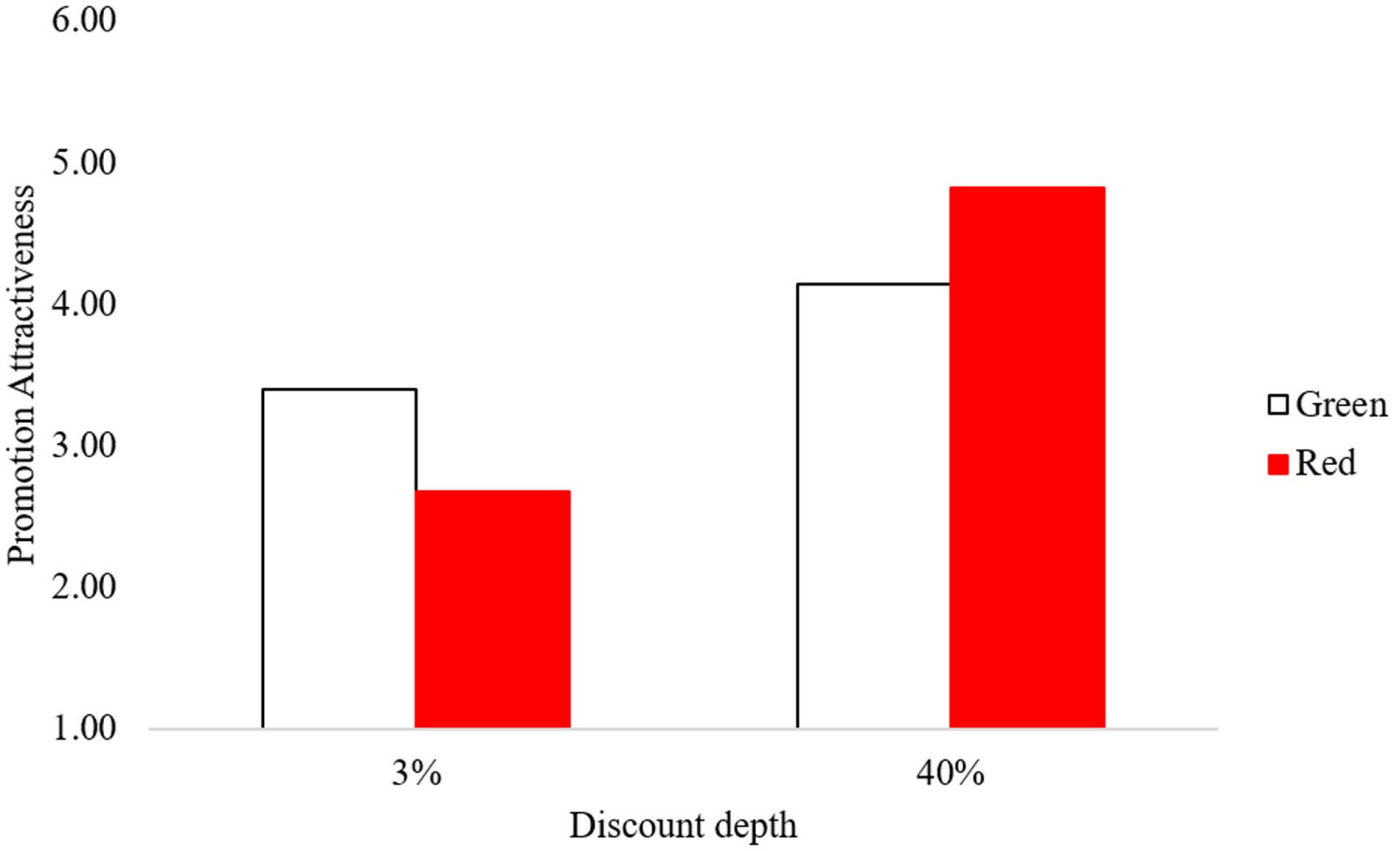
Promotion attractiveness as a function of font color and discount depth.

We conducted a two-way analysis of variance (ANOVA) including the font color of the price presentation and discount depth. Consistent with findings in Study 1, the analysis revealed a significant main effect of the discount depth (*F*(1,119) = 37.13, *p* < 0.01) on the perceived attractiveness to the advertised deal. In addition, this analysis yielded a highly significant two-way interaction between the font color of the price presentation and discount depth (*F*(1,119) = 8.85, *p* < 0.01). Next, we conducted planned contrast analyses. Consistent with our expectation, when the discount rate was high (40%), the perceived value of the advertised price promotion was higher with the red font (*M* = 4.82, *SD* = 1.07) versus the green font (*M* = 4.14, *SD* = 1.38), (*F*(1,119) = 3.96, *p* = 0.05). Conversely, when the discount rate was low (3%), the perceived value of the advertised deal was lower with the red font (*M* = 2.68, *SD* = 11.09) versus the green font (*M* = 3.40, *SD* = 1.59), (*F*(1,119) = 4.94, *p* = 0.03; see Figure 2).

##### Processing fluency as a mediator

We expected that the interaction between the price discount depth and the font color of the price presentation on promotion attractiveness would be mediated by processing fluency. To test this hypothesis, a bootstrapped mediated moderation analysis was used to test the model (model 8; Hayes, 2012). The mediation effect of processing fluency [indirect effect = −0.41, 95% CI (−0.75, −0.15)] was significant, whereas the direct effect of the interaction between the font color and discount depth on perceived attractiveness was not significant (*b* = −0.13, *t* = −1.29, *p* = 0.20). Therefore, the observed effect was completely mediated by processing fluency (Zhao et al., 2010).

**Figure 3:**
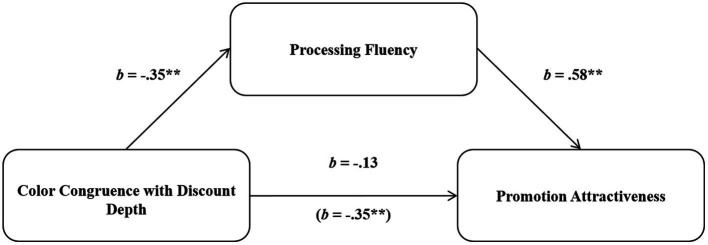
Research model and mediated moderation analysis.

Processing fluency was also confirmed as a meaningful mediator only in the advertising with the color red, which was associated with the price promotion [indirect effect = 0.41, 95% CI (0.21, 0.63)], but not in the advertising with the color green, which had no association with the price promotion [indirect effect = 0.00, 95% CI (−0.21, 0.21)]. Specifically, when the font color was red, processing fluency was greater in the high-discount condition (40%; *M* = 4.34, *SD* = 1.19) than in the low-discount condition (3%; *M* = 3.57, *SD* = 1.20), (*F*(1,119) = 6.44, *p* = 0.01). However, the mediating role of processing fluency was not observed in the green font color condition (*M_40%_* = 4.12, *SD* = 1.24 vs. *M_3%_* = 4.34, *SD* = 1.16), (*F*(1,119) < 0.54, *p* = 0.46; see Figure 3). These results provide rigorous evidence supporting processing fluency as a crucial mediator.

#### Discussion

The results of Study 2 replicated the findings from Study 1. In the high-discount condition, promotion attractiveness was higher for the advertisement with the red font than the green font for the price presentation, whereas in the low-discount condition, it was lower in the red font condition than the green font condition. The mediation test confirmed that the observed effect was driven by the experience of processing fluency.

However, there is still another possibility that the participants’ responses may reflect their previous persuasion knowledge of the use of color in price promotions, assuming that manufacturers may use red to maximize consumers’ value perception even when the actual savings is small. We tested this possibility in a post-hoc study using the same stimuli as in Study 2 (*n* = 69; 35 men and 34 women, M_age_ = 34.71). Persuasion knowledge was measured using four items on a 7-point scale (1 = strongly disagree, 7 = strongly agree). The items were: “The advertiser tries to excessively promote the value of the promotions,” “The advertiser tries to manipulate the audience in a way I do not like,” “I was annoyed by the advertisements above because the advertisers seem to inappropriately influence the audiences,” and “This ad was fair in what was said and shown” (Cronbach’s *α* = 0.72). We conducted a two-way ANOVA to test whether the participants’ persuasion knowledge may have played a mediating role. We found that the interaction effect of color and discount depth on persuasion knowledge was not significant (*F*(1,65) = 0.338, *p* = 0.563). This finding ruled out the possibility that the effects of Studies 1 and 2 can be attributed to persuasion knowledge.

## General discussion

Consumer price promotions are presented to consumers every day in all types of retail contexts. In many companies, more than half of their marketing budgets are spent on price promotions (Bolton et al., 2010; Nielsen, 2015; Bogomolova et al., 2017). The present research investigated if the common practices used in these price promotions, specifically the use of the color red in advertising, always help increase the attractiveness of the promotion. Across two empirical studies, we uncovered the boundary condition of the effect of the color red: the discount depth. Specifically, the effect of the color red in enhancing consumers’ value perception was only observed when people considered the degree of the discount to be moderate or high. When the offered discount was unambiguously low, the reverse pattern was shown: the use of red had a negative influence on perceived value. Moreover, the results of Study 2 revealed that congruence between the representation of the color red and discount depth enhanced processing fluency, which, in turn, led to perceived attractiveness to the promoted deal.

One may wonder if the level of readability depending on the different color combinations is the alternative explanation. According to previous literature, the contrast level of two colors positively influences the level of readability (Bruce and Foster, 1982; Clarke, 2002). That is, the contrast level can be a proxy for the level of readability. Based on this explanation, the contrast level of each color combination used in Studies 1 and 2 was calculated using a contrast checker.^1^ Based on the contrast checker, the contrast ratios are 3.99:1 for red text (RGB values = 255:0:0) on a white background, 2.86:1 for green text (RGB values = 0:176:80) on a white background (the control condition for Study 2), and 21:1 for black text on a white background (the control condition for Study 1). In sum, regardless of the contrast level of the color combination, the observed results in Studies 1 and 2 are consistent with our expectations. In other words, only the color red was associated with the savings, and the opposite pattern was shown for the other color conditions. Thus, we conclude that readability would not be a potential explanation in this context.

The current research contributes to the extant literature in several ways. First, this study adds to the literature on a congruency effect. Building on the increasing research on a congruency effect in advertising (Krishna et al., 2010; Fleck et al., 2012; Jang and Park, 2020; Wang et al., 2020), the current study highlights the importance of a match between non-central visual elements and the message. Specifically, we employ a commonly used color in price promotion advertising as an important source of a congruency effect that influences the attractiveness of the advertising. More importantly, our novel finding reveals a potential backfiring effect from the incongruence between the visual cues and the advertising content. This result expands the findings of prior literature suggesting a connection between the color and the message at a conceptual level.

Second, our work contributes to research that discusses the context-dependent nature of the meaning of color. Numerous researchers have investigated the conceptual associations of the color red under various domains such as hazards (De Bock et al., 2013) or warnings (Braun and Silver, 1995) and sexual connotations (Elliot and Niesta, 2008). However, only a handful of studies have investigated the meaning of red in a price promotion context (Puccinelli et al., 2013; Ye et al., 2020) despite retailers’ ubiquitous use of red in advertising. Our study provides additional evidence that the color red represents high value and savings. The strength of the association is further manifested by showing that the elicited associations may contrast with the actual discount depth of price promotions. Using red with a low discount depth could backfire and undermine the deal value and attractiveness.

Third, our work contributes to the literature on comparative price advertising. Studies in this area indicate that peripheral cues such as size (Coulter and Coulter, 2005), physical distance (Coulter and Norberg, 2009), and sound (Coulter and Coulter, 2010) can have differential effects on consumers’ perceptions of value depending on the discount depth. In particular, these studies have shown that a positive influence of non-diagnostic cues on price evaluation occurs only when the mental representation of the provided information (i.e., regular price, sale price, or discount depth) is compatible with the characteristics of non-diagnostic cues. The current findings extend the prior literature by bringing the color red to light as another contextual cue that moderates the effectiveness of comparative price advertising.

Finally, our findings contribute to the literature on processing fluency. Much research has demonstrated how the ease processing derived from the message-visual congruency influences people’s evaluations (e.g., Chae and Hoegg, 2013; Cian et al., 2015; Wang et al., 2020). Building on their research, our study demonstrates the interplay between the color and the message on consumer responses mediated by processing fluency. We also extend the literature demonstrating the influence of feeling-based judgments in a price promotion valuation context (Hsee and Rottenstreich, 2004; Strack and Deutsch, 2004; Park and Ryu, 2019; Jang and Park, 2020). Building on this stream of literature, we provide further evidence that subjective feelings from metacognitive experiences can play a crucial role even in tasks involving computational thinking such as a price promotion evaluation.

The current findings have obvious practical implications for marketers. Marketers attempt to use various means to highlight the value of their promotions, and using red is one of the most effective ways to signal a good value. However, when the value of the offer is significantly low, the use of red may lower the attractiveness of the promotion. More broadly, when a certain color has a strong association with a specific meaning, the color cues can facilitate the formation of consumers’ performance expectations for the products and services. These pre-set expectations may have a negative influence on satisfaction when the specific content does not meet those expectations. Therefore, colors should be considered carefully to ensure a match with what the target object is capable of offering.

Finally, the current study suggests potential avenues for further research. First, while a variety of promotional strategies can be used to deliver economic benefits to customers, the present work focuses on a price promotion context. Extending the findings to various types of promotions may be interesting. For example, gift or bonus pack promotions are often advertised with the color red to enhance consumers’ value perception. We presume that for typical gift promotions, a backfire effect of red may not be observed as the value of the promotion is difficult to calculate. However, if consumers have a comparative reference in mind that can be quantified in value, possibly from comparable promotions from competitors or previous purchase experiences, a diverging effect of the color red may be observed.

Second, the present work examined the discount depth as a potential moderator, but other factors consumers frequently encounter can be tested as sources of a red color-congruence effect. One potential distinction that may influence consumers’ valuation is the type of retailer (e.g., a prestigious retailer vs. thrifty retailers; Yoon et al., 2014). Consumers may perceive that a red-hot deal is congruent with their image of thrifty retailers but less so with the image of prestigious retailers. Thus, a red color-congruence effect may be more pronounced for thrifty retailers.

Lastly, further research is warranted to find other colors that serve as comparative references in evaluating advertising content. For instance, the color green and energy efficiency may be an interesting candidate to replicate the proposed effect in the current article. Colors are ubiquitous in marketing, and marketers of brands and retailers frequently use colors with specific associations, expecting that a certain color will strengthen the attractiveness of their offerings. Further research to understand the nature and context in which a negative effect of colors occurs should provide useful guidelines for marketing practitioners.

## Data availability statement

The raw data supporting the conclusions of this article will be made available by the authors, without undue reservation.

## Ethics statement

Ethical review and approval was not required for the study on human participants in accordance with the local legislation and institutional requirements. The patients/participants provided their written informed consent to participate in this study.

## Author contributions

All authors listed have made a substantial, direct, and intellectual contribution to the work and approved it for publication.

## Funding

This publication was supported by Brunel University London.

## Conflict of interest

The authors declare that the research was conducted in the absence of any commercial or financial relationships that could be construed as a potential conflict of interest.

## Publisher’s note

All claims expressed in this article are solely those of the authors and do not necessarily represent those of their affiliated organizations, or those of the publisher, the editors and the reviewers. Any product that may be evaluated in this article, or claim that may be made by its manufacturer, is not guaranteed or endorsed by the publisher.

## Footnotes

^1^ https://webaim.org/resources/contrastchecker/
