# Supplementary material for: Disadvantages of red: The color congruence effect in comparative price advertising
Source: Front Psychol. 2022 Nov 17;13:1019163. doi: 10.3389/fpsyg.2022.1019163 (PMC9712978; doi:10.3389/fpsyg.2022.1019163)
Supplement: Supplementary file 1 [file Table_1.DOCX]

**Appendices**

Appendix A. Stimuli for Study 1

|  | | Background color | | |
| --- | --- | --- | --- | --- |
|  |  | Red | White | Blue |
| Discount depth | High | 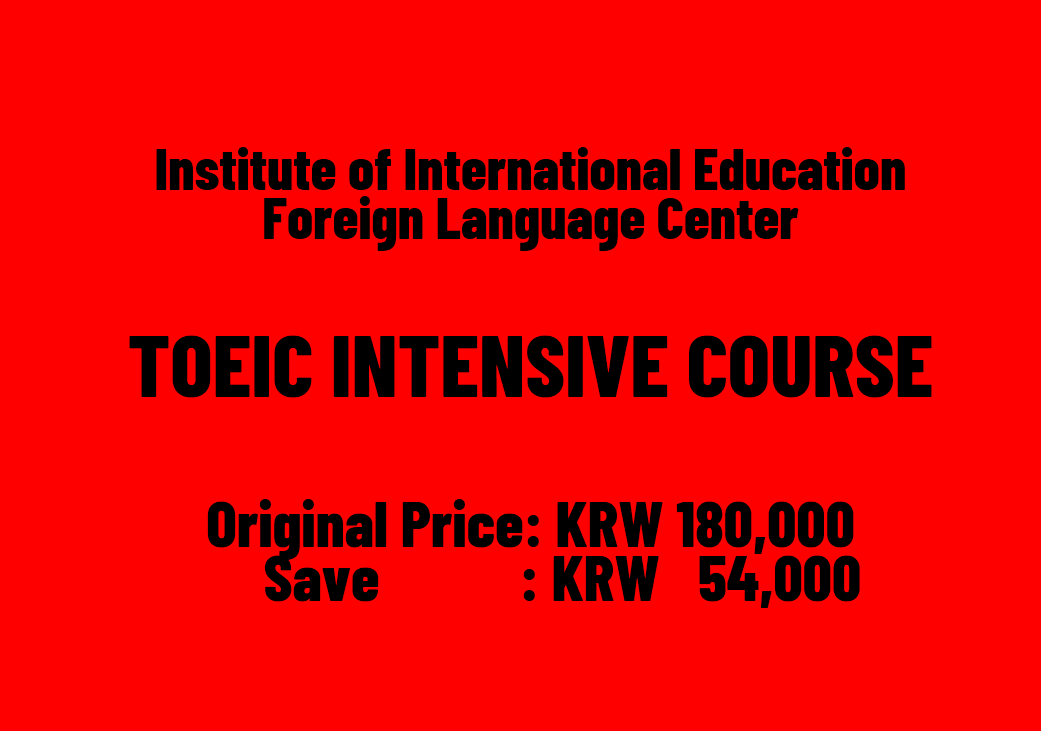 | 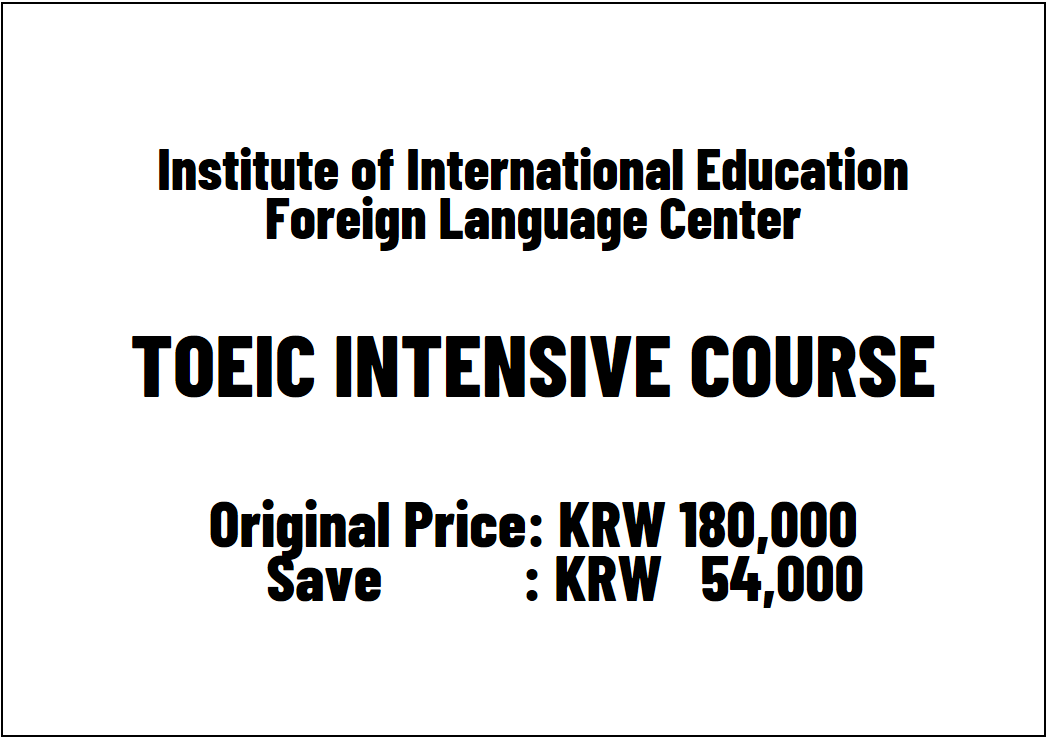 | 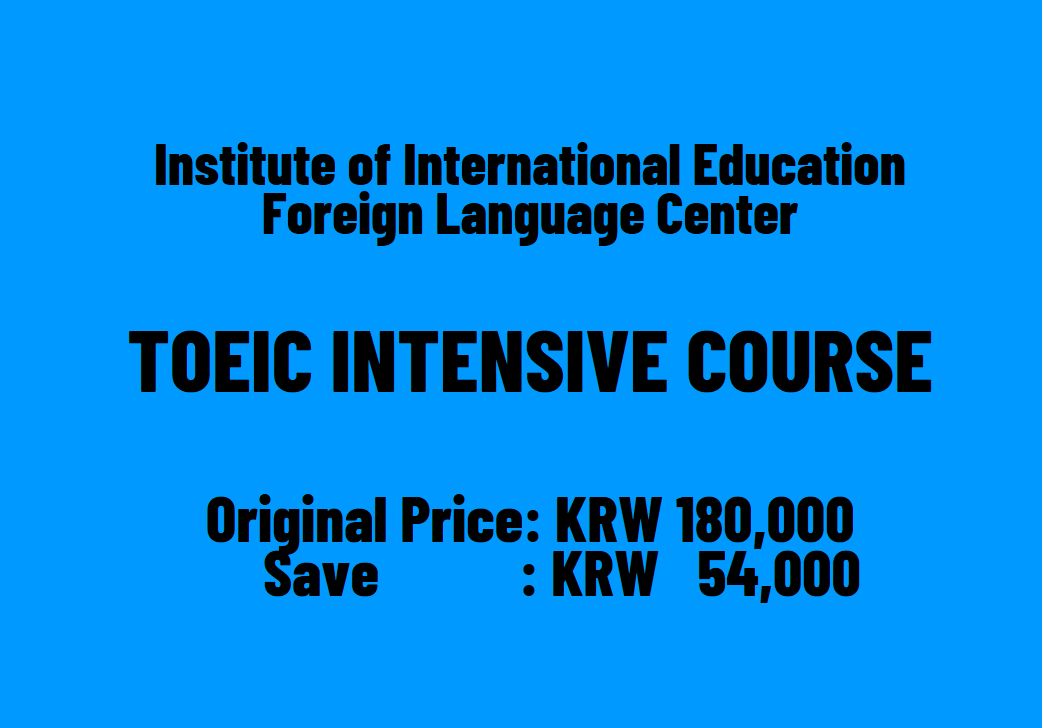 |
|  | Low | 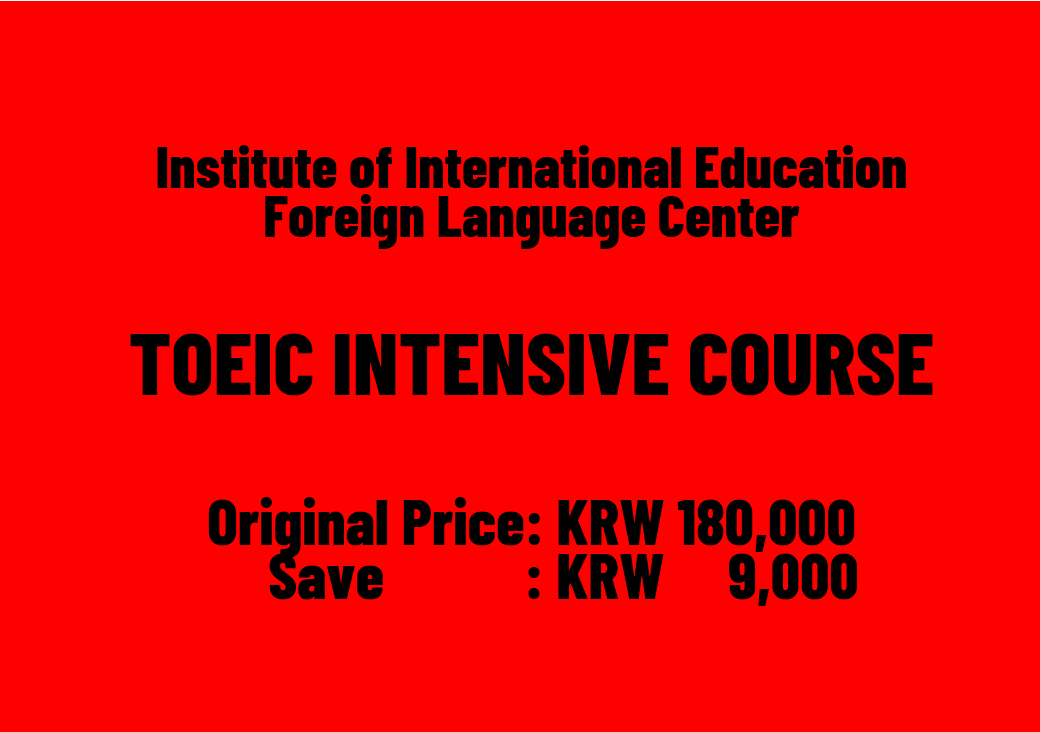 | 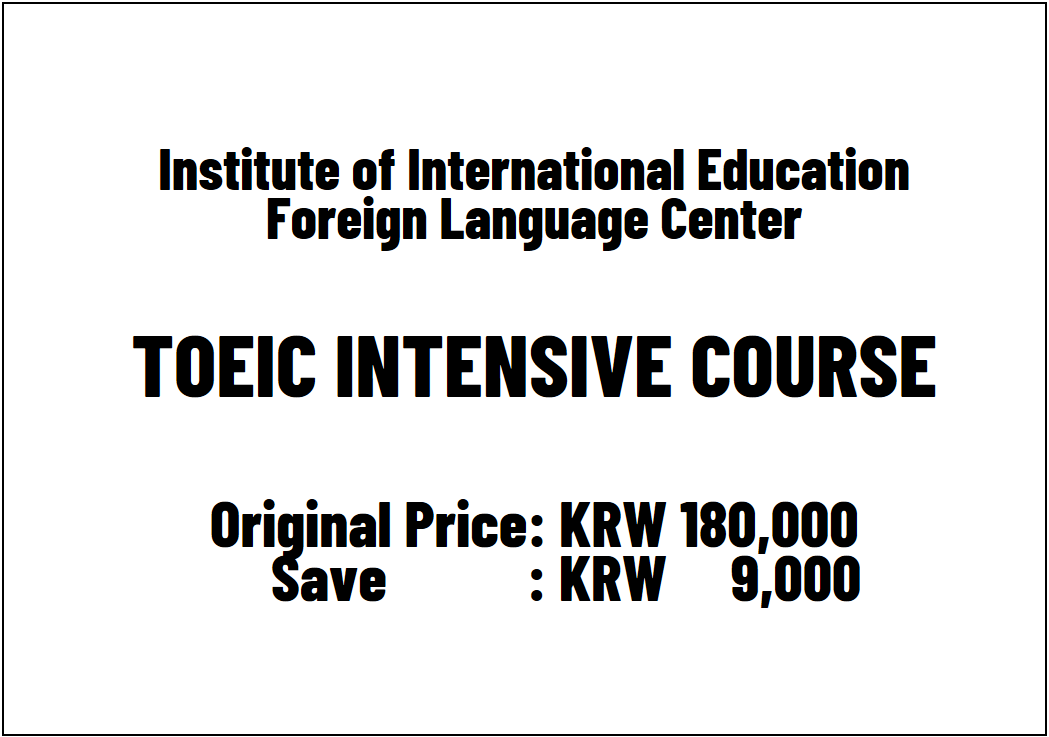 | 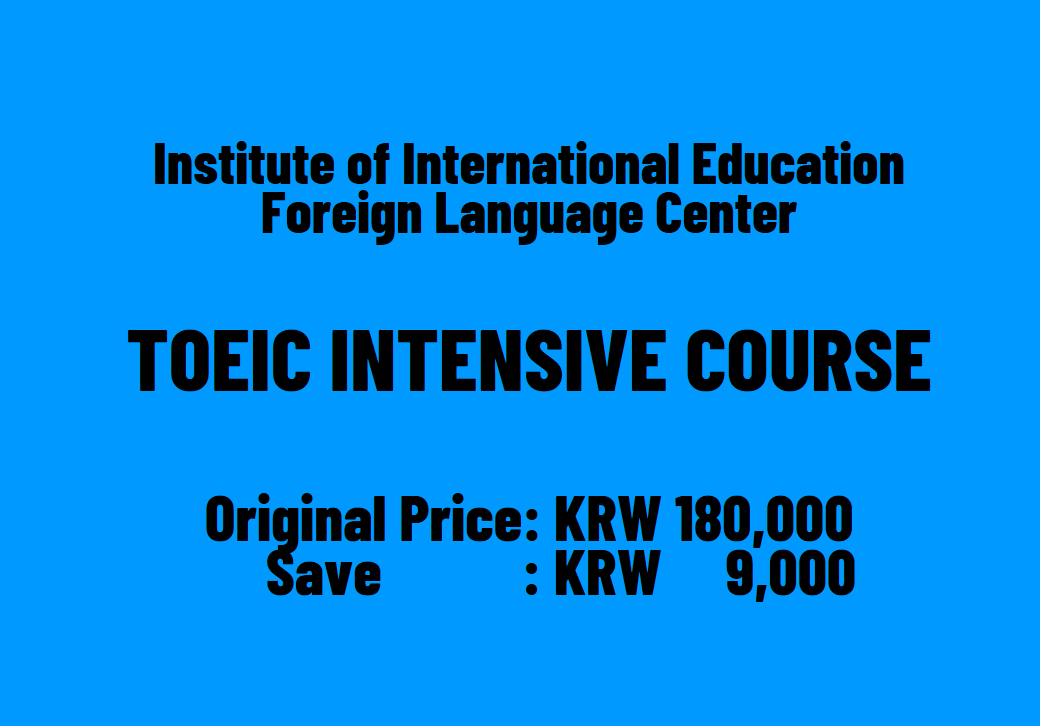 |

Appendix B. Stimuli for Study 2

|  | | Font color | |
| --- | --- | --- | --- |
|  |  | Red | Green |
| Discount depth | High | 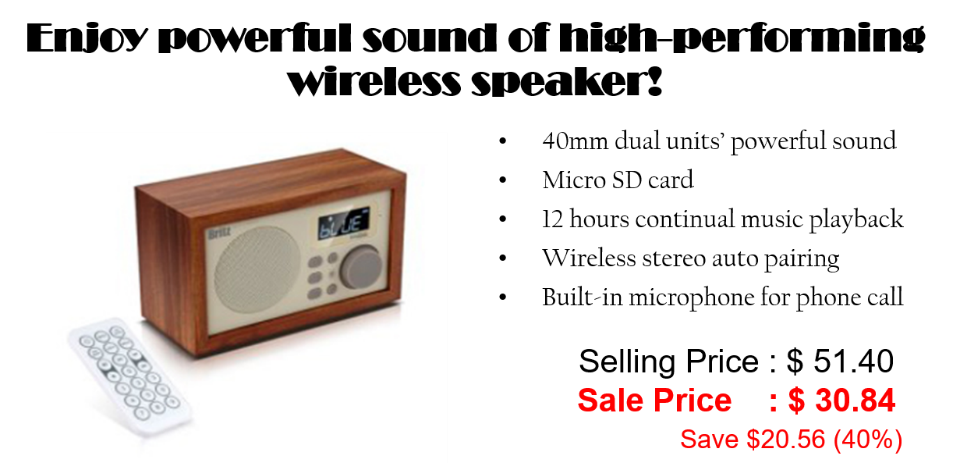 | 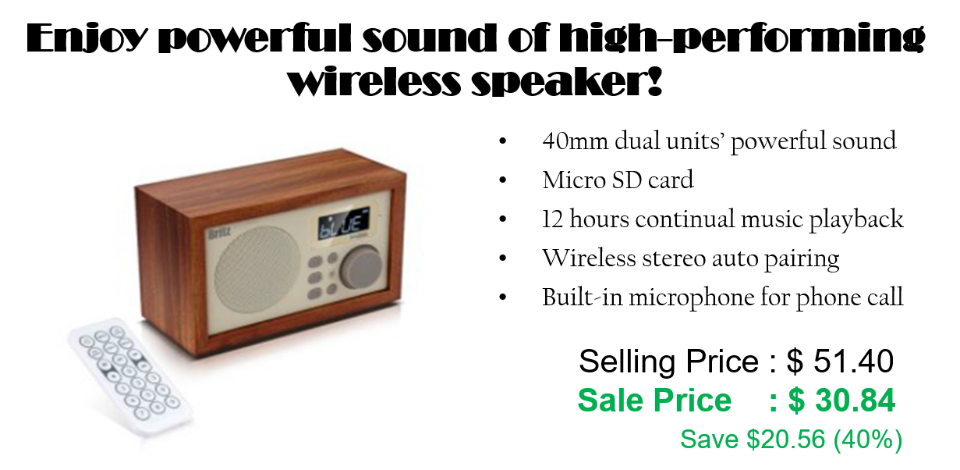 |
|  | Low | 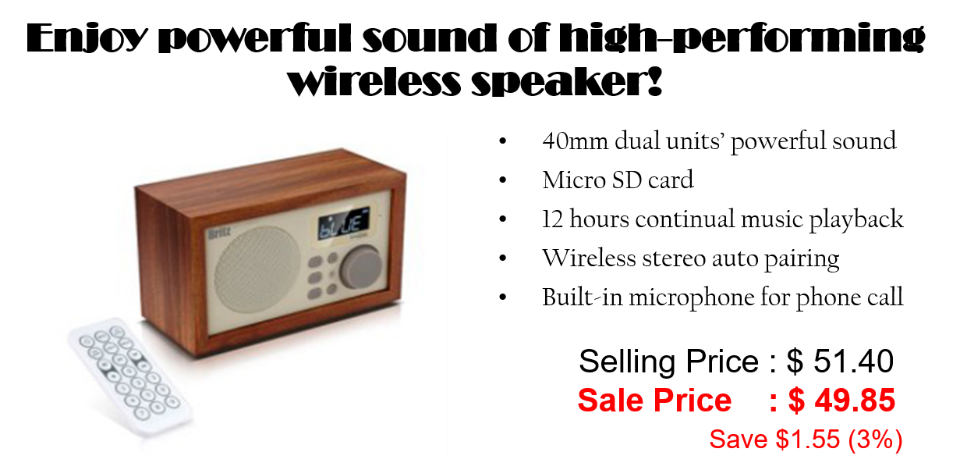 | 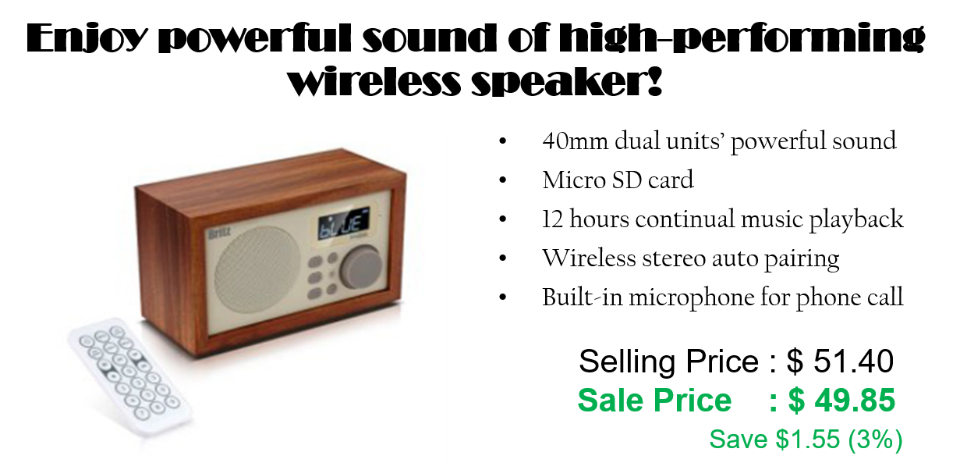 |
